# Crochetage, the Forgotten Electrocardiographic Sign

**DOI:** 10.7759/cureus.46498

**Published:** 2023-10-04

**Authors:** Carol Fernandez Hazim, Mohammed Shaban, Dessiree Cordero, Ana P Urena Neme, Miguel A Rodriguez Guerra

**Affiliations:** 1 Medicine, Montefiore Medical Center, Albert Einstein College of Medicine, New York, USA; 2 Internal Medicine, BronxCare Hospital Center, Icahn School of Medicine at Mt. Sinai, New York, USA; 3 Cardiology, Medicina Cardiovascular Asociada, Santo Domingo, DOM

**Keywords:** pediatric cardiology, crochetage sign, third-degree atrioventricular block, pacemaker placement, patent foramen ovale closure

## Abstract

Acquired complete heart block is a rare but severe arrhythmia caused by various factors such as infections, medications, and autoimmune conditions. Atrial septal defect (ASD) is a common congenital heart defect, with larger ASDs possibly causing symptoms such as fatigue, shortness of breath, and frequent respiratory infections. In some cases, high-grade atrioventricular block with ASD can occur; however, the exact incidence is not well established. We report a rare case of a 15-year-old male presenting with acute dizziness. Initial electrocardiogram (EKG) showed a complete heart block with a Crochetage sign. Patent foramen ovale (PFO) was confirmed by transthoracic and transesophageal echocardiograms. Closure of PFO and permanent pacemaker resulted in complete resolutions of symptoms and disappearance of Crochetage sign.

## Introduction

Atrial septal defect (ASD) is one of the most common types of congenital heart disease (CHD), with an incidence of 6-10% [1,2]. Despite its high incidence, the subtle physical findings, and in many cases, the absence of symptoms, it might be undiagnosed. Numerous cases, usually young adults, remain unknown until complications arise [3]. Echocardiography may be limited; therefore, a bedside electrocardiogram (ECG) can provide clues to its existence. A distinct but often-unrecognized pattern to indicate the presence of an ASD on the ECG is the Crochetage sign [4]. This sign is a notch appearing at the rising edge or peak of the R wave in the inferior limb leads [2]. In 1996, Heller et al. correlated the Crochetage pattern in at least one inferior lead of 73% of patients with a secundum atrial septal defect [5]. Recognizing this pattern can improve patient management and speed care. We describe a young patient who presented at the emergency department after a sudden-onset dizziness episode. His initial electrocardiogram revealed a third-degree atrioventricular (AV) block and the presence of the Crochetage sign.

## Case presentation

A 15-year-old male with no past medical history was brought into the emergency department after an episode of sudden-onset dizziness. The episode occurred while he was sitting during a class at school, and the school nurse noted that his heart rate was in the low 40s. He denied chest pain, shortness of breath, or diaphoresis. Notably, two weeks before the presentation, he had a symptomatic coronavirus disease 2019 (COVID-19) infection with cough, nasal congestion, and fatigue.

**Figure 1:**
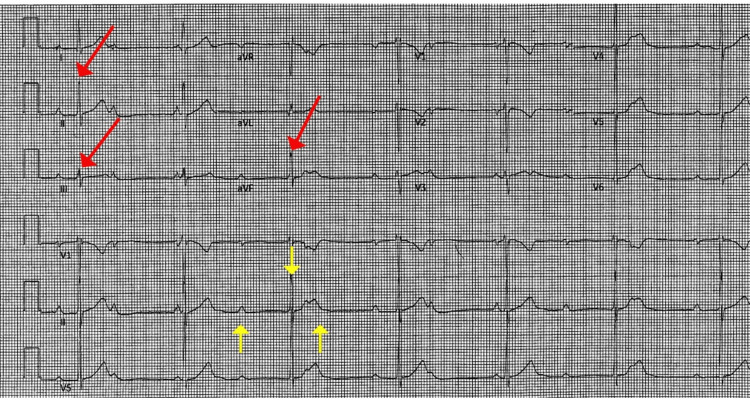
ECG reveals third-degree atrioventricular block (yellow arrows) and R wave notching in the inferior leads (red arrows).

**Figure 2:**
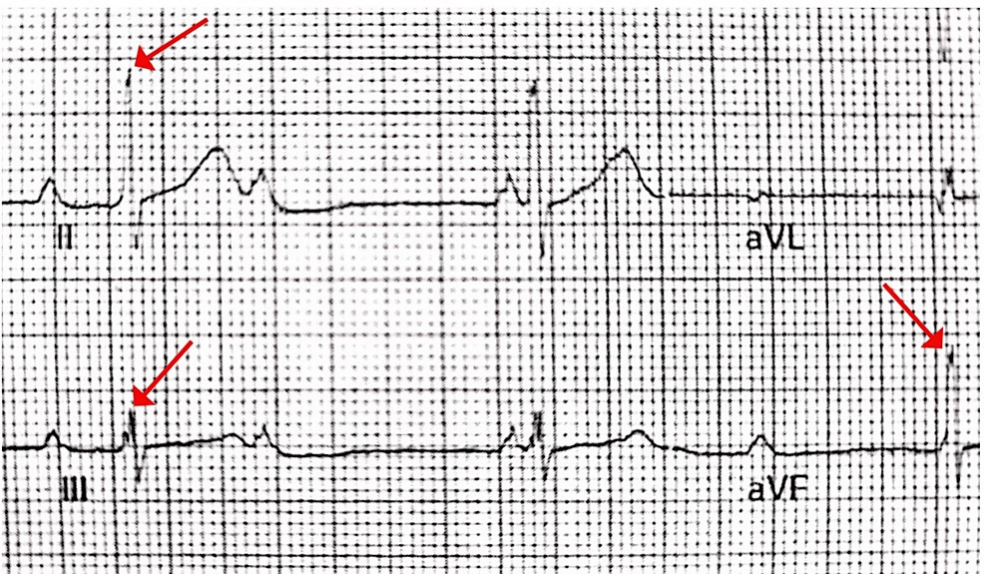
Crochetage sign with a notch near the apex of the R wave of inferior leads (arrows). Zoomed image from Figure 1

Initial vital signs were a respiratory rate of 17 breaths per minute, a heart rate of <40 beats per minute, a temperature of 37°C, and a blood pressure of 153/80 mmHg. Cardiac auscultation revealed a systolic murmur. The remainder of the physical examination was unremarkable. The initial ECG showed a third-degree AV block (Figure 1) and an R wave notching in the inferior leads (Figure 2). Laboratory workup was remarkable for troponin I 1.16 ng/mL (<0.03), C-reactive protein 2.89 mg/dL (<0.8); chemistry, complete blood count, and B-natriuretic peptide within normal limits.

The patient was transferred to a tertiary care center and admitted to the pediatric intensive care unit. Subsequently, he was started on isoproterenol and theophylline. Nevertheless, he did not show evidence of AV node function recovery in the following days. For suspected COVID-19 myocarditis, and while pending cardiac magnetic resonance imaging (MRI) results, he was started on steroids to decrease inflammation at the AV node and on ceftriaxone for empiric therapy for possible Lyme carditis.

Eventually, the patient did not recover AV node function; hence was planned for pacemaker placement. Cardiac MRI showed no evidence of myocardial edema or inflammation and no myocardial late gadolinium enhancement. The transthoracic echocardiogram (TTE) imaging was limited in assessing the atrial septum. However, it revealed evidence of a right-to-left shunt with the saline contrast injection. Therefore, he underwent transesophageal echocardiography (TEE) evaluation before the planned pacemaker procedure. TEE confirmed tunneled patent foramen ovale (PFO) with left-to-right shunting. The team successfully performed a transcatheter patent foramen ovale closure with a septal occluder device, followed by a transvenous dual chamber pacemaker placement.

**Figure 3:**
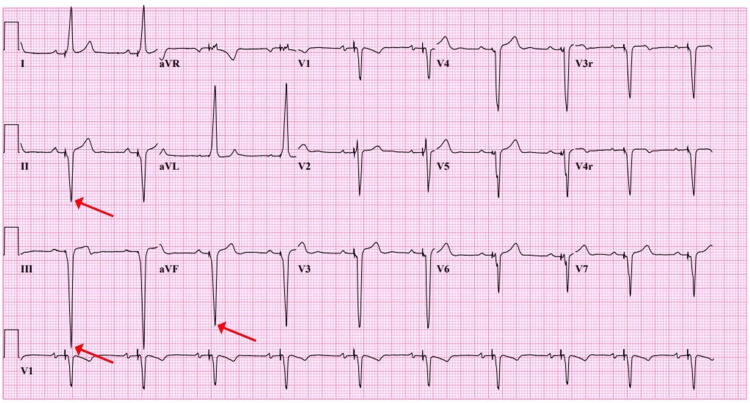
Disappearance of Crochetage sign following patent foramen ovale (PFO) device closure (arrows).

The patient was discharged on aspirin 81 mg for six months. He reported the resolution of his symptoms on subsequent outpatient follow-ups. Furthermore, a follow-up ECG demonstrated the complete disappearance of the Crochetage sign in the inferior leads (Figure 3).

## Discussion

CHD may not be diagnosed until later in life, and its incidence can vary depending on several factors, including genetics, the definition of CHD used, and the diagnostic methods used [6,7]. ASD is a common congenital heart defect (CHF) and is more common in females than males [8,9]. The majority of ASDs are small and do not cause significant health problems. However, larger ASDs may lead to complications such as pulmonary hypertension or heart failure [9]. The incidence of high-grade AV-block with ASD is relatively rare. Overall, acquired complete heart block is rare. Symptoms of complete heart block in children can vary depending on the severity of the condition. Mild cases may not cause symptoms, while more severe cases can cause dizziness, fainting, shortness of breath, chest pain, and fatigue [10].

The exact incidence of heart block with ASD is not well established. Still, it is estimated to be less than 1% following the device closure and surgical repair of the ostium secundum [8]. AV block and patients with ASD could also be related to a genetic mutation in myocardial transcription factors such as NKX2.6, GATA5 and TBX5. These mutations could be associated with familiar types of ASD with variable degrees of AV block, such as Holt-Oram syndrome. Extracardiac skeletal manifestations such as absent radial bone and polydactyly can also be present in those cases [11].

The Crochetage sign is an EKG pattern with a small notch or "bump" near the apex of the R wave in the inferior leads (II, III, aVF). This sign is also known as the "notch sign" or "the slur" [12]. The term "crochetage" comes from the French word for "hook," and the sinus pattern resembles a hook or crochet-like shape. This sign is most commonly seen in patients with ASD, although it can also be seen in other conditions such as PFO, pulmonary embolism, right ventricular hypertrophy, and patent ductus arteriosus [13,14]. This sign has been associated with the severity of the shunt and has been suggested as an ECG marker of a PFO associated with ischemic embolic stroke [4].

The mechanism behind the Crochetage sign is not fully understood, but it is thought to be related to the delayed depolarization of the right ventricle in patients with ASD. The notch represents a delay in the electrical impulse as it travels from the right ventricle to the left ventricle across the atrial septum [15]. The Crochetage sign has high specificity but relatively low sensitivity for detecting ASD [12,14]. In other words, the Crochetage sign can be a useful diagnostic tool in identifying ASD. However, it should be interpreted with other clinical and diagnostic findings. Diagnostic tests, such as echocardiography and cardiac MR, are needed to confirm the diagnosis.

The management of ASD in childhood is guided by clinical guidelines developed by the American Heart Association (AHA) and the European Society of Cardiology (ESC) [16,17]. According to the AHA guidelines, children with ASD may require intervention if they have symptoms such as shortness of breath, fatigue, or exercise intolerance. Intervention may also be needed for large or moderate-sized defects (>6 mm diameter) that cause right ventricular volume overload or pulmonary hypertension. The presence of paradoxical embolism, a rare complication that can lead to stroke or other thromboembolic events, or associated defects such as ventricular septal defects may also indicate a need for intervention [16].

Both the AHA and ESC guidelines recommend consideration of transcatheter closure as the preferred treatment modality for most children with ASD, particularly for secundum ASDs that are less than 2 cm in diameter and not associated with other defects. Surgical closure may be recommended for larger ASDs or those not amenable to transcatheter closure [16,17]. The treatment of CHB in adolescents depends on the underlying cause of the block, the presence of symptoms, the degree of heart block, or its permanency [18]. In cases of permanent CHB, treatment may involve using a pacemaker to maintain adequate heart rate and rhythm [19].

The Crochetage sign disappears or decreases in amplitude following PFO closure, which is thought to be due to the elimination of the right-to-left shunt that occurs in patients with PFO, which leads to a more uniform and synchronous depolarization of the right and left ventricles [14,20]. Therefore, the disappearance of the Crochetage sign following PFO closure may be a useful indicator of the successful closure of the defect [13].

In summary, acquired complete heart block is most commonly caused by infections, certain medications, autoimmune conditions or CHD [10]. The data about the difference in the incidence of Crochetage in ASD vs. PFO is heterogeneous. In our patient the CHB may be caused by his current viral illness. However, this electrocardiographic sign was specific for his PFO. This finding reinforces the association of this ECG sign and structural heart disease, highlighting the importance of identifying this inferior leads notch or in the R wave. 

## Conclusions

In the era of emergent advanced cardiac imaging, such as echocardiography, cardiac MRI, and nuclear imaging, EKG findings are almost out of clinical significance in managing childhood CHD. However, a “notch” in the R wave, known as the Crochetage sign, can lead the clinician toward a possible differential diagnosis of ASD. Further testing and possible treatment options, such as the closure of the PFO, providing the resolution of symptoms and avoiding potential complications, including heart failure and cerebrovascular accidents.
